# Clinicopathological evaluation of anoxic mucosal injury in strangulation ileus

**DOI:** 10.1186/1471-2482-14-79

**Published:** 2014-10-16

**Authors:** Ryuji Takahashi, Yoshito Akagi, Takaho Tanaka, Atsushi Kaibara, Sugako Kajiwara, Ichirou Shima, Jun Taguchi, Tomoaki Mizobe, Tatsuyuki Kakuma, Kazuo Shirouzu

**Affiliations:** 1 Department of Surgery, Kurume University School of Medicine, Kurume, Japan; 2 Department of Surgery, Social Insurance Tagawa Hospital, Tagawa, Japan; 3 Department of Pathology, Social Insurance Tagawa Hospital, Tagawa, Japan; 4 Department of Surgery, Asakura Medical Association Hospital, Asakura, Japan; 5 Department of Pathology, Asakura Medical Association Hospital, Asakura, Japan; 6 Department of Biostatistics Center, Kurume University, Kurume, Japan

**Keywords:** Strangulation ileus, Anoxic injury, Mucosal injury, Creatine kinase, Base excess

## Abstract

**Background:**

In patients with strangulation ileus, the severity of bowel ischemia is unpredictable before surgery. To consider a grading scale of anoxic damage, we evaluated the pathological findings and investigated predictive factors for bowel gangrene.

**Methods:**

We assessed 49 patients with strangulation ileus who underwent a laparotomy between January 2004 and November 2012. Laboratory tests and the contrast computed tomography (CT) were evaluated before surgery. According to the degree of mucosal degeneration, we classified anoxic damages into the following 3 grades. Ggrade 1 shows mild mucosal degeneration with extended subepithelial space. Grade 2 shows moderate degeneration and mucosal deciduation with residual mucosa on the muscularis mucosae. Grade 3 shows severe degeneration and mucosal digestion with disintegration of lamina propria.

**Results:**

Resected bowel specimens were obtained from the 36 patients with severe ischemia, while the remaining 13 patients avoided bowel resection. The mucosal injury showed grade 1 in 11 cases, grade 2 in 10 cases, and grade 3 in 15 cases. The patients were divided into two groups. One group included grade 1 and non-resected patients (n = 24) while the other included grades 2 and 3 (n = 25). When comparing the clinical findings for these groups, elevated creatine kinase (*P* = 0.017), a low base excess (*P* = 0.021), and decreased bowel enhancement on the contrast CT (*P* = 0.001) were associated with severe mucosal injury.

**Conclusion:**

In strangulation ileus, anoxic mucosal injury progresses gradually after rapid spreading of bowel congestion. Before surgical intervention, creatine kinase, base excess, and bowel enhancement on the contrast CT could indicate the severity of anoxic damage. These biomarkers could be the predictor for bowel resection before surgery.

## Background

Strangulation ileus is a critical condition leading to emergency surgeries, in which patients require early diagnosis and surgical decision making. Even when an immediate laparotomy is performed, many patients must undergo a bowel resection due to severe anoxic injury. To avoid undesirable outcomes for the patients, the clinicians must detect bowel strangulation as soon as possible using diagnostic equipment. Although previous studies have investigated several diagnostic parameters for strangulation ileus [1-6], the severity of bowel ischemia remains to be unpredictable before surgery. To consider a grading scale of anoxic injury, we evaluated the pathological findings in resected bowel specimens and investigated predictive factors for bowel gangrene.

## Methods

### Study design and obtained approval

This study was retrospective observational study based on the medical records. We concentrated consecutive patients with strangulation ileus who underwent a laparotomy at two hospitals in Japan affiliated with Kurume University School of Medicine (Social Insurance Tagawa Hospital, Tagawa, and Asakura Medical Association Hospital, Asakura) between January 2004 and November 2012. Resected bowel specimens were obtained from a total of 36 patients. Blood samples were obtained from a total of 49 patients. Subsequently, we evaluated laboratory data and contrast computed tomography (CT) findings. This observational study was approved by the local ethics committees (The Ethics Committee of Social Insurance Tagawa Hospital, Tagawa, and The Ethics Committee of Asakura Medical Association Hospital, Asakura) and patients had given written informed consent for both the treatment and publication of the data.

### Patient eligibility and data acquisition

We assessed 49 patients with strangulation ileus who underwent a laparotomy at the two hospitals. In all cases, the strangulated small bowel was discovered during surgery. Each patient received a physical examination, laboratory tests, and a contrast computed tomography (CT) before surgery. Laboratory tests, including a white blood cell (WBC), absolute neutrophil count, C-reactive protein (CRP), lactate dehydrogenase (LDH), creatine kinase (CK), pH and base excess in blood gas analysis, were conducted within 24 hours prior to the operation. The absolute neutrophil count was calculated by multiplying the WBC by the cell frequency in the visual observation method using May-Grünwald-Giemsa stain. In addition, we assessed the contrast CT finding, time to surgery from onset time, duration of hospitalization, and postoperative complication. Onset time is the time at which the patient began to feel acute abdominal pain.

### Pathological evaluation of resected bowel specimens

**Figure 1:**
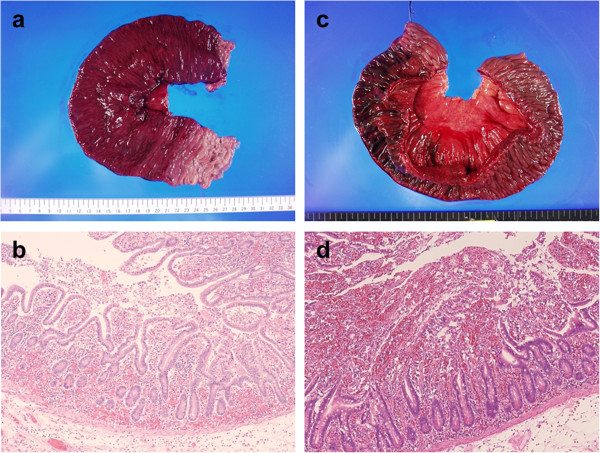
**Macro**- **and microscopic findings of grades 1 and 2. a** The mucosa of grade 1 shows a segmental color change to deep red. **b** The mucosal injury of grade 1 shows mild mucosal degeneration with extended subepithelial space (H & E stain, ×10). **c** The mucosa of grade 2 shows a segmental color change to reddish brown. **d** The mucosal injury of grade 2 shows moderate degeneration and mucosal deciduation with residual mucosa on the muscularis mucosae (H & E stain, ×10).

**Figure 2:**
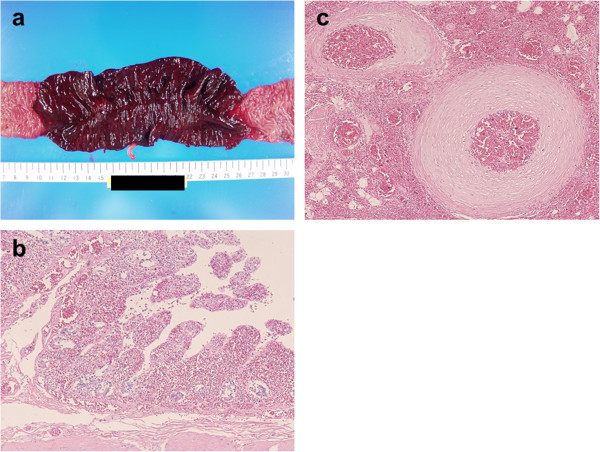
**Macro- and microscopic findings of grade 3**, **and fibromuscular intimal thickening of the vasa recta. a** The mucosa of grade 3 shows a segmental color change to dark red. **b** The mucosal injury of grade 3 shows severe degeneration and mucosal digestion with disintegration of lamina propria (H & E stain, ×10). **c** The microscopic finding shows fibromuscular intimal thickening of the vasa recta without occlusive thrombus (H & E stain, ×10).

To evaluate the degree of anoxic injury, we assessed resected bowel specimens (Hematoxylin and eosin stain; H & E stain). Transversally resected specimens were obtained from the strangulated bowel. Pathological findings were evaluated by the first author of this manuscript and a pathological specialist in hepatology and digestive disease. First, we observed the anoxic changes in bowel specimens pathologically and noticed the degree of mucosal degeneration in the mucosal layer. Then we classified the severity of ischemic injury into 3 grades according to the degree of mucosal degeneration. Our grading scale is compatible with a previous report [7] which categorizes ischemic changes of the mucosal lesion into 6 grades. Our grade 1 shows mild mucosal degeneration with extended subepithelial space. Grade 2 shows moderate degeneration and mucosal deciduation with residual mucosa on the muscularis mucosae. Grade 3 shows severe degeneration and mucosal digestion with disintegration of lamina propria. Macro- and microscopic findings of these mucosal degenerations are shown in Figures 1 and 2 [Figure 1a and b show mild degeneration (grade 1); Figure 1c and d show moderate degeneration (grade 2); Figure 2a and b show severe degeneration (grade 3)]. When the inhomogeneity of these mucosal damages was observed, the most severe damage was administrated.

### Statistical analysis

According to the degree of mucosal injury and surgical procedure, the patients were divided into two groups. Patients’ characteristics, laboratory data, and radiographic findings for the two groups were compared using the Mann–Whitney U test for continuous variables and the Fisher-Freeman-Halton exact test for categorical variables, respectively. All tests were two-sided and *P* values less than 0.05 were considered to be significant. Statistical tests were performed using JMP version 10 (SAS Institute Inc., Cary, NC, USA) and StatXact version 8 (Cytel Inc. Cambridge, MA, USA).

## Results

### Patients’ overall characteristics

The patients’ overall characteristics are shown in Table 1. Eighteen male and 31 female patients were enrolled in this study. Their median age was 77 year-old and median body mass index (BMI) was 19. The causes of bowel strangulation were ileus bands (42), internal hernias (4), and volvuluses (3). Forty patients had at least a history of laparotomy and details of the operations were as follows, gynecologic surgeries (14), appendectomies (9), gastrectomies (7), colectomies (5), cholecystectomies (3), surgical repairs for obturator hernias (3), low anterior resections (2), enterolyses (2), urological surgeries (2), partial bowel resection for traumatic perforation (1), ventriculoperitoneal shunting (1), and a Y-graft replacement (1). Although these operations included 15 oncological surgeries, neither cancerous ileus nor peritoneal carcinomatosa was discovered during surgery. On the physical examination, a sign of peritoneal irritation was shown in 23 patients before surgery.

**Table 1:** **Patients**’ **overall characteristics** (**n** = **49**)

**Character**	**Data or No. of patients**
Age	77 (38–96)
Gender	
Male	18
Female	31
BMI	19 (14–30)
Cause of bowel strangulation	
Ileus band	42
Internal hernia	4
Volvulus	3
History of laparotomy (Y/N)	40 / 9
History of oncological surgery (Y/N)	15 / 34
Peritoneal irritation sign (Y/N)	23 / 26
Time to surgery from onset time (hours)	21 (3–77)
Duration of hospitalization (days)	19 (7–121)
Postoperative complication (Y/N)	20 / 29
Hospital death (Y/N)	2 / 47

Values are median (range) or No. of patients; values in parentheses are ranges. *BMI*, body mass index; (Y/N), (Yes/No).

### Pathological findings of anoxic injuries

A total of 36 bowel specimens (H & E stain) were evaluated pathologically. The anoxic mucosal injury showed mild degeneration in 11 cases, moderate degeneration in 10 cases, and in 15 cases, the degeneration was severe. Four of the 10 cases with moderate degeneration showed inhomogeneity of mucosal damages. For these 4 cases, we categorized the mucosal injury according to the severest finding. The other common pathological findings showed congestion and/or hemorrhage (3 mild, 9 moderate, and 24 severe), and neutrophil and/or lymphocyte infiltrations (10 mild, 14 moderate, and 12 severe). Four of the 36 cases showed fibromuscular intimal thickening of the vasa recta without occlusive thrombus (shown in Figure 2c).

### Operative methods and postoperative complications

All patients underwent a laparotomy under general anesthesia. At the time of laparotomy, the site of strangulation marks was successfully detected in all patients. In the cases requiring a bowel resection, we performed a partial or massive resection of the small bowel with a concurrent end-to-end anastomosis. The median length of resected bowel was 70 cm (range, 20–250). The median length of resected bowel was longer in grades 2 and 3 patients than grade 1 patients (120 cm, 100 cm, and 50 cm, respectively; *P* = 0.006) In the cases not requiring bowel resection, we only released the bowel strangulation. The median period of hospitalization was 19 days. During hospitalization, 20 patients experienced postoperative complications. The complications included surgical site infections (8), pneumonitis (7), postoperative ileus (3), liver failure (2), disseminated intravascular coagulation [DIC] (2), short bowel syndrome (1), respiratory failure (1), and renal failure (1). There was no patient who developed anastomotic leakage or bowel perforation after surgery. We lost 2 patients during hospitalization from acute renal failure and aspiration pneumonitis, respectively.

### Comparisons of patients’ characteristics and laboratory data

As we previously mentioned, the patients were divided into two groups. One group was defined as non-gangrene group (NG group) which included grade 1 and non-resected patients (n = 24). The other group was defined as bowel gangrene group (BG group) which included grades 2 and 3 patients (n = 25). Comparisons of patients’ characteristics for these groups are shown in Table 2. The NG group included a higher proportion of male patients than those in the BG group (*P* = 0.019). There were no significant statistical differences associated with age, BMI, history of laparotomy, history of oncological surgery, peritoneal irritation sign, time to surgery from onset time, postoperative complication, duration of hospitalization, or hospital death event. Furthermore, comparisons of laboratory data for these groups are shown in Table 3. There were no significant statistical differences in the WBC, absolute neutrophil count, CRP, LDH, or pH between the two groups. However, higher levels of CK and lower levels of base excess were observed in the BG group (Wilcoxon test, *P* = 0.017 and 0.021, respectively). The mean levels of CK showed 90 IU/l in the NG group and 197 IU/l in the BG group, respectively. The mean levels of base excess showed 0.52 mEq/l in the NG group and −1.84 mEq/l in the BG group, respectively. Gender was not a confounder because levels of CK and base excess were not associated with gender (Wilcoxon test, *P* = 0.987 and 0.717, respectively).

**Table 2:** **Comparisons of patients**’ **characteristics for the NG group and BG group**

**Character**	**Non**-**gangrene group (n = 24)**	**Bowel gangrene group (n = 25)**	***P***
Age	77 (51–94)	74 (50–96)	0.756*
Gender			0.019
Male	13	5	
Female	11	20	
Cause of strangulation			0.598†
Ileus band	22	20	
Internal hernia	1	3	
Volvulus	1	2	
History of laparotomy (Y/N)	20 / 4	20 / 5	0.567
History of oncological surgery (Y/N)	10 / 14	5 / 20	0.096
Peritoneal irritation sign (Y/N)	10 / 14	13 / 12	0.571
Time to surgery from onset time (hours)	16 (3–74)	19 (5–77)	0.851
Postoperative complication (Y/N)	8 / 16	11 / 14	0.561
Duration of hospital stay (days)	18 (9–118)	18 (20–121)	0.233
Hospital death (Y/N)	1 / 23	1 / 24	0.235

Values are median (range) or No. of patients; values in parentheses are ranges. (Y/N), (Yes/No). *A Wilcoxon test. †A Fisher-Freeman-Halton exact test. The other analyses are performed by Fisher’s exact tests.

**Table 3:** Comparisons of laboratory data for the NG group and BG group

**Laboratory parameter**	**Non**-**gangrene group (n = 24)**	**Bowel gangrene group (n = 25)**	***P***
WBC	11200 (6264)	13625 (6227)	0.119
Absolute neutrophil count	9540 (6326)	12124 (5955)	0.089
CRP	2.78 (5.46)	5.36 (8.19)	0.303
LDH	239 (62)	252 (110)	0.984
CK	90 (71)	197 (132)	0.017
pH	7.44 (0.15)	7.43 (0.09)	0.562
Base excess	0.52 (5.72)	−1.84 (5.02)	0.021

Values are mean (standard deviation); values in parentheses are standard deviations. *WBC*, white blood cell; *CRP*, C-reactive protein; *LDH*, lactate dehydrogenase; *CK*, creatine kinase. All analyses are performed by Wilcoxon tests.

### Contrast CT findings and diagnostic accuracy

The common contrast CT findings for the NG group and BG group are shown in Table 4. The most common CT finding was ascites which was shown in 44 cases, while we detected peritoneal free air in 4 cases and hepatic portal gas in 1 case. The contrast CT finding showed mesenteric congestion in 31 cases, closed bowel loop in 28 cases, and decreased bowel enhancement in 22 cases, respectively. Consequently, 36 patients were diagnosed with strangulation ileus by CT scans, while the remaining 13 patients had initially been diagnosed with adhesive ileus. These 13 patients had an ileus tube inserted upon admission and subsequently underwent a laboratory, because their fluoroscopy findings showed irremediable bowel stenosis. Statistical analyses of these radiographic findings showed that decreased bowel enhancement on the contrast CT (Fisher’s exact test, *P* = 0.001) was associated with severe mucosal injury. Gender was not a confounder because decreased bowel enhancement on the contrast CT was not associated with gender (Fisher’s exact test, *P* = 1.000).

**Table 4:** Comparisons of contrast CT findings for the NG group and BG group

**Contrast CT finding**	**Non-gangrene group (n = 24)**	**Bowel gangrene group (n = 25)**	***P***
Mesenteric congestion	15	16	1.000
Closed bowel loop	15	13	0.567
Decreased bowel enhancement	5	17	0.001
Ascites	20	24	0.190

Values are No. of patients. All analyses are performed by Fisher’s exact tests.

### Cases presentation

**Figure 3:**
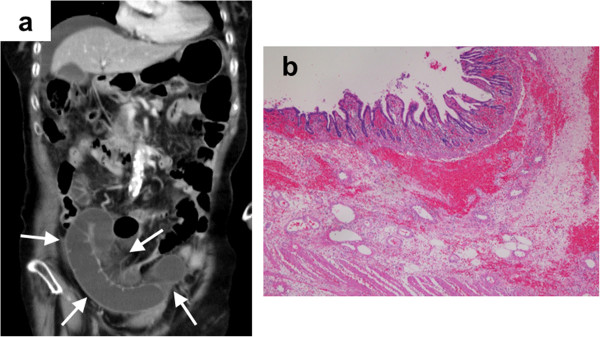
**Radiographic and pathological findings of the first case. a** The contrast CT shows closed bowel loop, mesenteric congestion, and mild ascites (*arrows*). **b** Pathological findings show grade 1 mucosal injury, moderate congestion and hemorrhage (H&E stain, ×4).

**Figure 4:**
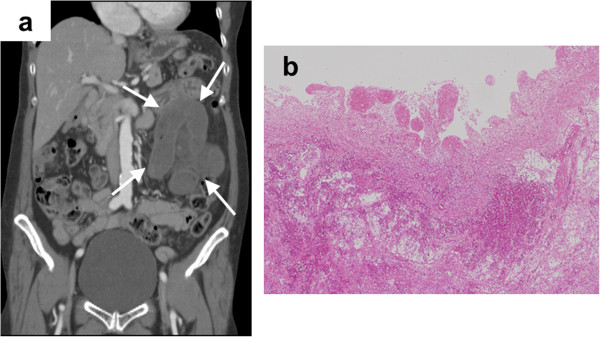
**Radiographic and pathological findings of the second case. a** The contrast CT shows decreased bowel enhancement, closed bowel loop, mesenteric congestion, and mild ascites (*arrows*). **b** Pathological findings showed grade 3 mucosal injury, severe congestion and hemorrhage (H&E stain, ×4).

Here we introduce the two patients who underwent a massive resection of the small bowel. The first case is a 61-year-old man who visited our hospital after 4 hours from onset time. Levels of CK and base excess showed normal range before surgery, and the contrast CT showed closed bowel loop, mesenteric congestion, and mild ascites (shown in Figure 3a). We resected 60 cm of his small bowel and pathological findings showed grade 1 mucosal injury, moderate congestion and hemorrhage (shown in Figure 3b). The second case is a 72-year-old woman who visited our hospital after 18 hours from onset time. In this case, a low base excess (−4.5 mEq/l) and elevated CK (378 IU/l) were observed before surgery, and the contrast CT showed decreased bowel enhancement, closed bowel loop, mesenteric congestion, and mild ascites (shown in Figure 4a). We resected 195 cm of her small bowel and pathological findings showed grade 3 mucosal injury, severe congestion and hemorrhage (shown in Figure 4b).

## Discussion

Can we evaluate the severity of anoxic injury in patients with strangulation ileus? We propose a grading scale for ischemic mucosal injury, which Chiu et al initially discovered in the dogs [7]. They categorized ischemic changes of the mucosal lesion into 6 grades (graded from 0 to 5); grade 0 − normal mucosal villi; grade 1 − development of subepithelial Gruenhagen’s space; Grade 2 − extension of the subepithelial space with moderate lifting of epithelial layer from the lamina propria; Grade 3 − massive epithelial lifting down the sides of villi; Grade 4 − denuded villi with lamina propria and dilated capillaries exposed; Grade 5 − digestion and disintegration of lamina propria. They confirmed that the speed of mucosal injury depended on arterial blood flow of the small bowel. Our grading scale of mucosal injury corresponded to each grade of their criteria, such as our grade 1 to their grade 0 – 2, our grade 2 to their grade 3 – 4, and our grade 3 to their grade 5, respectively.

To predict bowel strangulation and its severity, which radiographic finding is helpful? Our evaluation of contrast CT scans found that mesenteric congestion, closed bowel loop, and decreased bowel enhancement are helpful in discovering bowel strangulation. A meta-analysis of 15 separate studies found CT scans to be 83% sensitive and 92% specific in detecting an ischemic bowel in patients with obstructive ileus [8]. Sheedy et al found that decreased segmental enhancement was the most specific sign for small bowel ischemia [9]. Hayakawa et al mentioned that the direct CT findings (without contrast) suggestive bowel strangulation include high-density bowel wall, mesenteric congestion, and localized pneumatosis, while the indirect CT findings include C- or U-shaped loops with mesenteric vessels converging toward the obstruction site, ascites, target sign, two adjacent collapsed round loops, and whirl sign [10]. Our results suggested that a decrease in bowel enhancement is associated with the degree of mucosal injury. The other diagnostic approaches include ascites analysis by abdominocentesis and laparoscopic surgery/adhesiolysis for selected patients with small bowel strangulation [11,12].

After making a decision to do a laparotomy, the surgeon must release the strangulated bowel and decide whether to resect or not. The decision regarding bowel resection or conservation is made based on the surgeon’s impression of the ischemic damage and viability. Therefore, it is very important to understand the progression of anoxic injuries after the strangulation. Sullins et al compared pathological changes for the arteriovenous and venous occlusions in horses with small bowel strangulation [13]. After 1 to 3 hour intervals, segments of arteriovenous occlusion became dark without edema and hemorrhage, while segments of venous occlusion were characterized by progressive congestion, edema, and hemorrhage especially in the mucosal layer. Park et al confirmed that ischemic periods of more than 20 minutes caused villous injuries and extensive tissue injury in rats, but there was no exacerbation of tissue injury after the reperfusion [14]. Otherwise, an isolated arterial occlusion of 40 to 60 minutes induced significant exacerbation of tissue injuries at reperfusion [14]. Although these animal experiments indicate that the mesenteric arterial occlusion rapidly induces exacerbation of tissue injury and necrosis, only few studies have evaluated pathological findings of strangulated bowel and non-occlusive mesenteric ischemia (NOMI) in humans [15-19]. Pathologically, intimal thickening in the vasa recta and arteritis without organic vascular occlusion are associated with the development of NOMI [19]. In our study, 4 of the 36 cases (11%) showed fibromuscular intimal thickening of the vasa recta. Generally, NOMI accounts for 20 − 30% of all cases of acute mesenteric ischemia with a mortality rate of nearly 50% [20]. One German article analyzed 62 patients with NOMI and concluded that leukocytosis, elevated serum lactate, and increased CK/CK-MB levels are specific parameters to diagnose NOMI [21]. Since we did not investigate serum lactate and CK-MB levels in our patients, further investigations should be performed to evaluate these biomarkers in strangulation ileus.

Ischemic or hemorrhagic bowel wall infarction results in anoxia and necrosis, providing ideal conditions for bacteria growth and multiplication [22]. Tissue damages progress with progressive mucosal epithelial cell slough, collagen disruption, edema, neutrophil infiltration of submucosa and muscular layers and serosa [23]. Bowel gangrene, caused by these types of tissue damage, promotes anaerobic acidosis and lactate production [6,24]. Furthermore, smooth muscle cells degenerate and eventually disrupt leading to the release of CK and its isoenzymes. Graeber et al reported that levels of serum CK and each of its isoenzymes became significantly elevated in dogs with strangulated infarction [25]. Despite the fact that we have to perform surgery as soon as possible, there was no correlation between time to surgery from onset time of symptom and bowel gangrene. Since the progression of anoxic damage showed many variations, time to surgery from onset time could not be a reliable predictor for bowel gangrene. Although our study evaluated a limited number of patients and blood samples at different times from onset, elevated CK and a low base excess which indicates the degree of acidosis could predict severe mucosal injury. These biomarkers and decreased bowel enhancement on the contrast CT could indicate the severity of anoxic mucosal injury in patients with strangulation ileus.

## Conclusion

In strangulation ileus, anoxic mucosal injury progresses gradually after rapid spreading of bowel congestion. Before surgical intervention, elevated CK, a low base excess, and decreased bowel enhancement on the contrast CT could indicate the severity of anoxic damage. These biomarkers could be the predictor for bowel resection before surgery.

## Abbreviations

WBC: White blood cell; CRP: C-reactive protein; LDH: Lactate dehydrogenase; CK: Creatine kinase; CT: Computed tomography; H & E: Hematoxylin and eosin; BMI: Body mass index; DIC: Disseminated intravascular coagulation; NG: Non-gangrene; BG: Bowel gangrene; NOMI: Non-occlusive mesenteric ischemia.

## Competing interests

*Disclosure*: The authors declare that they have no competing interests.

## Authors’ contributions

RT, TT and JT are responsible for the conception and design of the study, the acquisition, analysis and interpretation of data, and drafting the work. YA is responsible for the interpretation of data and drafting the work. AK, IS, TM and KS are responsible for the interpretation of data and revising the work critically. SK and TK are responsible for the acquisition and analysis of data and revising the work critically. All authors read and approved the final manuscript.

## Pre-publication history

The pre-publication history for this paper can be accessed here:

http://www.biomedcentral.com/1471-2482/14/79/prepub
